# Scabies Outbreak in Pediatric Populations of Bangladesh: A Perspective on Therapeutic Management, Risk Factors, and Public Health Implications

**DOI:** 10.1002/hsr2.72450

**Published:** 2026-04-27

**Authors:** Hemayet Hossain, Md. Al Muktadir, Snigdha Sharmin Binte Sayeed, Sojib Ahmed, Md. Hasan Ali, Khadiza Akter Brishty, Md. Shahidur Rahman Chowdhury, Md. Mahfujur Rahman

**Affiliations:** ^1^ Department of Veterinary Science and Animal Husbandry Teesta University Rangpur Bangladesh; ^2^ Department of Microbiology and Hygiene Bangladesh Agricultural University Mymensingh Bangladesh; ^3^ Department of Pathology Sylhet Agricultural University Sylhet Bangladesh; ^4^ Faculty of Veterinary, Animal and Biomedical Sciences Sylhet Agricultural University Sylhet Bangladesh; ^5^ Department of Zoology (GSSC) University of Dhaka Dhaka Bangladesh; ^6^ Department of Medicine Sylhet Agricultural University Sylhet Bangladesh

**Keywords:** Bangladesh, neglected tropical diseases, pediatric scabies, public health response, risk factors, scabies outbreak

## Abstract

**Background and Aims:**

Scabies remains a major cause of morbidity among Bangladeshi children, particularly in overcrowded and resource‐limited settings. This perspective synthesizes current evidence to contextualize the surge of pediatric scabies outbreaks reported across Bangladesh in 2025. This perspective aimed to map outbreak hotspots, identify key risk determinants, and highlight health system gaps in surveillance and diagnosis, and therapeutic management of scabies.

**Methods:**

Evidence was compiled from published literature, national health data, WHO reports, media alerts, interviews with 50 registered dermatologists, and ArcGIS‐based visualization of affected regions.

**Results:**

Outbreaks were widespread across urban slums, religious school (Madrasa), Rohingya refugee camps, university dormitories, flood‐affected districts, rural communities and multiple districts including Cumilla, Barisal, Noakhali, Rajshahi, Sylhet, Khulna, Mymensingh, and Cox's Bazar driven by poverty, overcrowding, poor sanitation, seasonal humidity, and limited access to dermatologic care. Treatment practices reported by surveyed physicians revealed that 5% permethrin cream remains the first‐line therapy, while oral ivermectin is increasingly used for severe, recurrent outbreaks, although availability and affordability remain inconsistent across districts. Reinfection rates in institutional settings and the absence of national surveillance systems further complicate control efforts.

**Conclusion:**

This perspective emphasizes the urgent need for integrated strategies, including strengthened surveillance, improved treatment access, community‐based hygiene interventions, and alignment with WHO's NTD roadmap. Strengthening these components is essential to reduce pediatric morbidity, prevent complications, and enhance Bangladesh's progress toward effective scabies control.

## 1 Introduction

The World Health Organization (WHO) has classified scabies as a neglected tropical disease (NTD) that affects over 200 million people globally from *Sarcoptes scabiei* var. *hominis* at any given time [1]. The burden of this disease is the greatest among the resource‐poor and the tropical regions, where the prevalence rate among children is between 5% and 50% [2]. Similarly, the latest research has affirmed that the occurrence of scabies is the most common in those areas that are densely populated and where the environment is humid [3, 4].

Children get exposed to scabies at a greater rate than adults because they come in contact with each other more frequently and share bedclothes, which leads to high levels of transmission [5]. School‐going children of low‐income countries often act as reservoirs of transmission for scabies and continue the spread of the disease through their homes and schools [6]. The psychosocial implications of scabies are significant because the sleep schedule gets interrupted due to the night‐time itching, which leads to reduced school performance [7]. If left untreated, children are at risk of developing secondary bacterial infections that might potentially cause complications such as glomerulonephritis or rheumatic heart disease [8].

Bangladesh is home to over 170 million people who live in a densely populated environment facing many socio‐economic difficulties [9]. The community‐based prevalence rate among children is between 6% and 12% whereas the rate is higher in urban slum and religious school environments [10]. Several districts in Bangladesh have reported recurrent scabies outbreaks (Dhaka, Mymenshingh, Chattogram, Comilla etc.) over the past few years that have primarily affected overcrowded, impoverished, and institutional settings, indicating broad susceptibility throughout the country [11, 12, 13, 14, 15, 16, 17]. In addition, children who sustain the highest burden of scabies face long‐term consequences that perpetuate cycles of poverty and ill health [18].

Therefore, addressing scabies among this population is essential to minimize morbidity, improve quality of life, and help Bangladesh reach the WHO's neglected tropical disease targets [19, 20, 21]. The objective of this perspective was to analyze scabies outbreaks and their therapeutic management among children in Bangladesh, identify significant risk factors associated with these outbreaks, and discuss the public health implications.

## 2 Sources of Evidence

**Figure 1:**
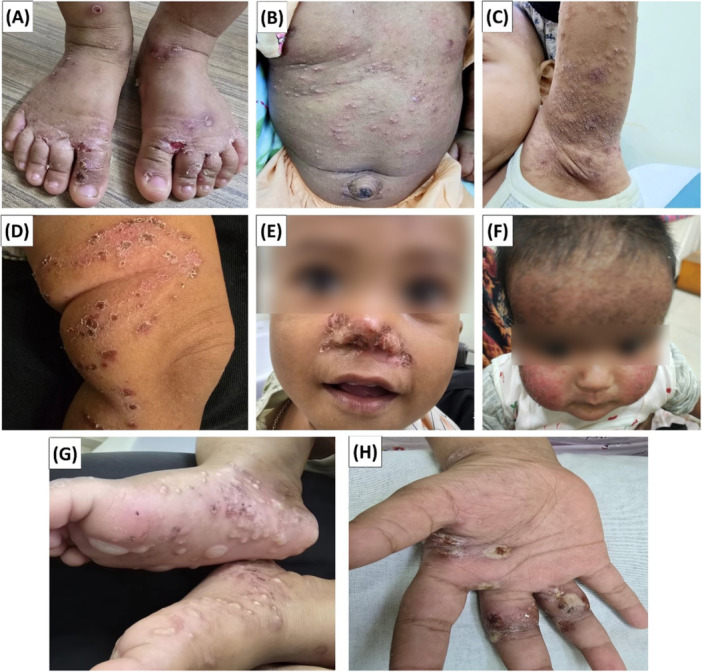
Clinical manifestations of scabies among children in Bangladesh. (A) Scabies with secondary bacterial infection on the feet and interdigital spaces; (B) Lesions around the umbilical area; (C) Scabies involvement in the axilla; (D) Lesions on the lower leg; (E) Crusted lesions around the nose; (F) Facial involvement with diffuse papular lesions; (G) Lesions on the plantar surface of the feet; (H) Lesions on the hand and interdigital areas.

According to the Directorate General of Health Services (DGHS) of Bangladesh, scabies prevalence among children in urban slums has risen in recent years, with outbreaks reported in Dhaka and Cox's Bazar Rohingya refugee camps. This perspective is based on secondary data for the identification of scabies outbreak in 2025. The data sources including published articles, government health statistics (http://nsds.bbs.gov.bd/en/topic/22/Health), DGHS Bangladesh official website (https://dghs.gov.bd/), WHO reports (https://www.who.int/bangladesh/health-topics), WHO Neglected Tropical Diseases fact sheets 2025 (https://www.who.int/publications/i/item/9789240114043), and outbreak coverage in Bangladeshi recognized media (The Daily Star, Prothom Alo, TV reports etc.). We conducted face‐to‐face interviews with 50 registered dermatologists across different scabies outbreak regions in Bangladesh to explore current therapeutic practices. Dermatologists were selected based on their active involvement in managing scabies cases in outbreak‐affected areas. Only participants who agreed to participate in the survey were included in the interviews. Finally, the outbreak regions were visualized on a map generated using ArcGIS software (ArcMap 10.8). The clinical manifestations of scabies among children in Bangladesh were depicted in Figure 1. Written consent was obtained from the parents for scientific use of the captured images, and all faces were blurred to ensure anonymity. This study was approved by Institutional Ethics Committee (IEC) of Teesta University, Bangladesh with an approval number: TU/IEC/2025/002.

## 3 Epidemiology and Ongoing Outbreaks of Scabies in 2025

**Figure 2:**
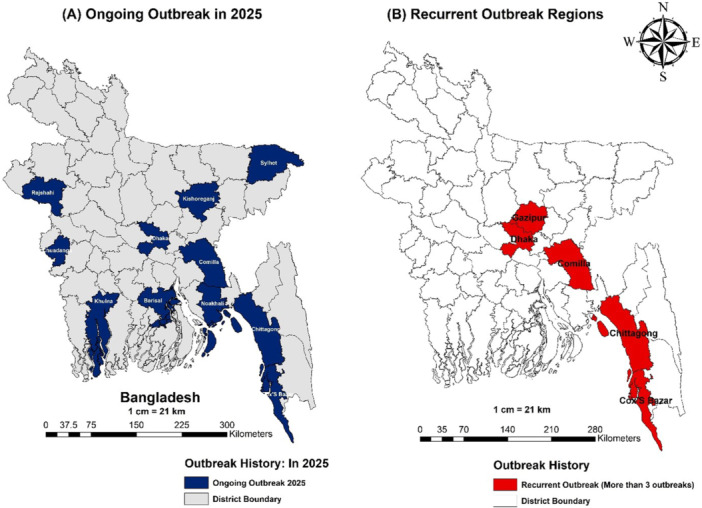
Geographic distribution of scabies outbreaks in Bangladesh. (A) Districts affected by the ongoing 2025 outbreak. (B) Districts identified as recurrent outbreak (More than 3 outbreaks) regions based on historical patterns.

Scabies is highly prevalent in Bangladesh, particularly among children under 15 years (6%–12%) (Table 1, Figure 2). Severe outbreaks have been reported in Cumilla, with up to 80% infection in dermatology patients and associated complications. Institutional spread is evident in Rajshahi University residential halls. Rural studies show 65% prevalence, with significant itching and sleep disturbance. Flood‐affected and slum areas, such as Barisal and Noakhali, report rapid transmission linked to overcrowding, displacement, and poor hygiene.

**Table 1:** Overview of reported scabies outbreaks in Bangladesh, 2025.

Outbreak hotspot and location	Settings	Affected group	Condition/Characteristics	References (media reports)
Cumilla (city and peri‐urban clinics)	Spread across rural and urban areas	Children and adults	80% of dermatology patients infected; kidney complications noted	[12]
Rajshahi University (residential halls)	Confined to dormitories	Adults (students)	Daily rise in cases; reinfection ~71%	[11]
Barisal (slum clusters)	Multiple slum blocks	Children and adults	80% prevalence; poverty and hygiene deficiency linked	[13]
Noakhali (dense community areas)	Abrupt multi‐ward spread	Children and adults	Sudden outbreak; shared sleeping mats accelerated transmission	[15]
Sylhet and Khulna (rural divisions)	Rural poor sanitation areas	Children	Outbreak linked to poverty and poor sanitation	[14]
Flood‐affected districts (north‐central Bangladesh)	Disaster shelters	Children and adults	2800 cases among 3500 patients in 2 days; displacement & poor sanitation	[22]
Chattogram (city and adjacent districts)	Spike across multiple thanas	Children and adults	Increased prevalence over a month; humid season worsened spread	[23]
Chuadanga (district towns)	Community transmission	Children and adults	Community transmission reported; rising cases in local clinics	[24]

Similarly, in the Cox's Bazar Rohingya Refugee Camps, Médecins Sans Frontières (MSF) has conducted approximately 179,000 consultations for scabies within the first 18 months of operation [25]. Khulna and Sylhet divisions have faced outbreaks of scabies, especially among populations that live in rural areas, where poverty and limited access to healthcare increase vulnerability [14]. In Mymensingh, records show an increasing prevalence of scabies in school‐going children, which reflects the disproportionate number of pediatric victims in rural villages [5, 16]. Cases are more frequent during humid monsoon months, when there is a rise in overcrowding in shelters, which creates opportunities for the parasite to transmit [3]. Based on the comparative data available, Bangladesh's prevalence is greater than that of many other South Asian countries, which show socioeconomic differences and environmental challenges [9]. Internationally, scabies affects more than 200 million people at any given time, positioning the burden of Bangladesh within a broader neglected tropical disease context [1].

## 4 Current Risk Factors Driving Outbreaks

### 4.1 Socioeconomic Determinants

In Bangladesh, scabies continues to spread largely because poverty and malnutrition weaken immune systems, curbing access to proper hygiene and making children more susceptible to both scabies and bacterial infections [10, 13]. Favorable environments for the mites to thrive were created by poor housing conditions and overcrowding, which facilitate skin contact and transmission [10, 14].

### 4.2 Environmental and Climatic Factors

The tropical climate of Bangladesh (high humidity and warm temperatures) accelerates sweating, and close contact in shelters creates the ideal conditions for the survival and transmission of mites [3, 9]. Flood worsens the transmission process by forcing the population into temporary shelters with poor sanitation [11]. Environmental struggles, along with poverty, significantly contribute to the lack of access to clean water and hygiene facilities for displaced families [17, 25].

### 4.3 Healthcare System Challenges

In the rural areas, dermatology services are restricted, which hampers timely diagnosis and treatment, resulting in many cases being untreated and misdiagnosed [16, 18]. However, late diagnosis, the shortage of supply of scabicidal medications in both rural clinics and refugee camps, make the disease further complicated to control [26]. Moreover, even after sufficient medications, the rate of reinfection remains high in institutional residential halls such as Rajshahi University [11]. The absence of national surveillance systems for scabies reflects broader gaps in neglected tropical disease control within Bangladesh [21].

### 4.4 Behavioral and Cultural Practices

In low‐earning households, it is a common practice to share bedding and clothing, which increases the risk of transmission among children [5, 8]. Close living setups and communal sleeping practices in religious schools (Madrasa) are related to high prevalence, with studies suggesting infection rates of 34% among students [10]. The sudden outbreak in Noakhali pointed out how behavioral practices like shared sleeping mats promote rapid transmission [7, 15].

## 5 Therapeutic Management

### 5.1 Topical Scabicides

Topical scabicides were the most commonly reported therapeutic approach for scabies management. Permethrin 5% cream was the most frequently used treatment (45 times), recommended by 90% of doctors across regions including Cumilla, Barisal, Noakhali, Sylhet, and Khulna, and was used for both children and adults (Table 2). Benzyl benzoate lotion was less commonly used (6%) in Sylhet and Khulna, mainly for children. Sulfur ointment was rarely used (4%), reported only in Mymensingh rural settings and prescribed exclusively for children. Overall, treatment practices varied by region and patient age group.

**Table 2:** Therapeutic practices for scabies management in Bangladesh (Survey of 50 dermatologists, 2025).

Types	Therapeutic group	Generic medicines (No. of times recommended)	Regions reporting use	% of doctor suggesting	Age/Group treated
Scabicides (principal therapeutics)	Topical scabicides	Permethrin 5% cream (45)	Cumilla, Barisal, Noakhali, Sylhet, Khulna	90%	Children and Adults
		Benzyl Benzoate lotion (3)	Sylhet, Khulna	6%	Children only
		Sulfur ointment (2)	Mymensingh (rural schools)	4%	Children only
Supportive therapeutics	Oral antiparasitics	Ivermectin (17)	Rajshahi University, Cox's Bazar, Noakhali	34%	Adults (institutional outbreaks) and Children (refugee camps)
	Antibiotics	Amoxicillin (7), Azithromycin (4)Erythromycin (5)AmoxicillinClavulanic Acid (3)	Cumilla, Flood‐affected districts	38%	Children and adults (secondary infections)
	Antihistamines	Pheniramine Maleate (22), Promethazine HCl (11), Chlorpheniramine(6), Diphenhydramine (3)	All surveyed regions	84%	Children and adults
	Supportive agents	Multivitamins (11), Vitamin B Complex (11), Vitamin C (4), Zinc (7), Selenium (2), Amino Acids (10)	Barisal, Sylhet, Khulna, Cox's Bazar	90%	Children and adults

### 5.2 Supportive Medications

Oral ivermectin is increasingly administered in severe, recurrent outbreaks particularly in university dormitories and refugee settlements although supply and affordability remain inconsistent. In children, alternative agents such as benzyl benzoate lotion and sulfur ointment are prescribed in settings where permethrin is unavailable. Secondary bacterial infections are frequently managed with antibiotics including amoxicillin, azithromycin, and erythromycin. Antihistamines remain essential for symptomatic relief of pruritus, while supportive supplements are commonly provided to enhance recovery and overall skin health.

## 6 Public Health Implications

Scabies inflicts a high burden on pediatric health services in Bangladesh, with high child caseloads in dermatology clinics [10]. In Cumilla, 80% of the patients needed extensive care [12]. Similarly, from the camps of Cox's Bazar, reported consultations totaled 179,000 over a period of 18 months. Current outbreaks in institutions such as Rajshahi University, with 70% recurrence, raise the need for repeated treatments [11].

The fact that scabies is one of the neglected tropical diseases suggests that it is important in the WHO 2021–2030 NTD strategy, hence requiring concerted effort [21]. With a high prevalence among children, Bangladesh is considered to bear the highest burden in South Asia and, as such, is an intervention priority while aligning with the global NTD goals [1, 9].

If left untreated, secondary infections such as impetigo and cellulitis can result from scabies infection, leading to various complications like post‐streptococcal glomerulonephritis and pediatric morbidity. Over 60% of the affected children suffered itching and sleep disruption in rural Bangladesh, highlighting its physical and psychosocial impact [6, 7, 17].

Repeated scabies treatments strain families financially and burden public health. In addition, outbreaks of this skin disease in slums and camps, coupled with limited supplies, increase costs and stress in healthcare [14, 18, 26].

## 7 Current Strategies and Gaps

In Bangladesh, scabies treatment primarily relies on topical permethrin for adults and children [18]. Oral ivermectin is used in some institutional and refugee settings but is limited by cost and availability [7]. Local products like Scabex Cream 5% are commonly prescribed, though irregular supply and affordability reduce effectiveness in low‐income communities [26]. Reinfection rates remain high, with 71% of treated Rajshahi University students affected, highlighting the need for ongoing interventions [11].

In our opinion, the over‐reliance on topical permethrin without ensuring adherence to the second application dose is a critical gap contributing to high reinfection rates.

School‐based campaigns teach hygiene and symptom recognition, reducing stigma and promoting timely treatment in high‐prevalence institutions [10, 14]. Rural health education is limited by resources and coordination [13], while targeted interventions in Cox's Bazar camps remain beneficial despite overcrowding [25].

Despite efforts, Bangladesh lacks mass drug administration policies effective in other endemic areas [1]. Surveillance is weak due to unstandardized scabies reporting, limiting outbreak response and resource allocation as well as low research funding leaves transmission and long‐term control poorly understood [21]. Without stronger policy commitment and integration into the national NTD agenda, scabies will continue to disproportionately affect children and vulnerable communities [17]. We suggest that the absence of a standardized national scabies reporting system reflects a systemic under‐prioritization of this disease within Bangladesh's public health agenda.

## 8 Adolescent‐Focused Scabies Control Measures

The current approach to scabies control in Bangladesh remains largely reactive rather than preventive, particularly in adolescent populations who act as key transmission reservoirs in schools, madrasas, and institutional settings. The reliance on individual treatment without synchronized management of close contacts significantly contributes to persistent reinfection cycles. We argue that scabies control strategies must transition toward a community‐based and adolescent‐focused intervention model. Specifically, periodic mass drug administration (MDA) using permethrin or ivermectin in high‐risk institutions, combined with mandatory treatment of household and dormitory contacts, could substantially reduce transmission. Additionally, adolescent‐targeted health education programs should be prioritized, as this age group plays a critical role in maintaining transmission networks through close physical interaction and shared living conditions. Digital health surveillance systems integrated with school health programs may allow early outbreak detection and rapid response. Importantly, behavioral compliance particularly adherence to repeated treatment regimens remains a major overlooked barrier in Bangladesh, necessitating simplified treatment protocols and supervised application strategies. In our view, without incorporating these targeted, system‐level interventions focusing on adolescents, existing efforts will continue to provide only temporary relief rather than sustainable scabies control.

## 9 Recommendations and Future Directions

### 9.1 Strengthening Surveillance

Scabies monitoring in Bangladesh needs to be more robust which can be a part of community‐based reporting within slums for timely detection [10], and integrating into the national NTD program as part of global strategies will facilitate Bangladesh at optimizing resources [21].

### 9.2 Improving Access to Treatment

Low‐cost and accessible scabicidal drugs are needed; the shortages of permethrin, ivermectin etc. hamper control of scabies in low‐income areas [18, 26]. Training rural healthcare workers and expanding diagnostics beyond cities can improve management and reduce treatment inequities [14, 16].

### 9.3 Preventive Measures

School hygiene promotion can minimize the risk of scabies through close contact and the sharing of bedding [5]. Again, awareness programs can promote early treatment and minimize the stigma associated with the condition [8]. The need for targeted interventions in most informal settlements, with Cox's Bazar being particularly overcrowded, recording 179,000 consultations within a period of 18 months, and a high prevalence recorded in the slums of Barisal at 80%, calls for effective action [13, 25].

### 9.4 Research Priorities

Future studies should explore prevalence differentiation between rural and urban settings, targeting children also [7, 25]. Climate change enhances outbreaks of scabies [3], in that case, seasonal epidemiological studies can help inform its prevention [17]. Increasing funding and integrating scabies into the nation's NTD agenda are crucial for the long‐term control of this disease [1].

## 10 Conclusion

The present perspective underscores that scabies remains an under‐recognized yet significant public health concern for Bangladeshi children, with recent outbreaks reflecting deep‐rooted socioeconomic, environmental, and health‐system vulnerabilities. The evidence compiled from national data, outbreak investigations, and physician insights highlights not only the widespread geographic distribution of cases in 2025 but also the systemic gaps that allow these outbreaks to persist. While permethrin and ivermectin constitute the backbone of current therapeutic practice, inconsistent availability, delayed diagnosis, and high reinfection rates continue to limit their effectiveness. Importantly, the findings illustrate that scabies control cannot rely solely on clinical treatment; rather, it requires coordinated improvements in surveillance, hygiene practices, community awareness, and integration into national NTD priorities. Addressing these challenges presents an opportunity for Bangladesh to reduce preventable morbidity and strengthen resilience against future outbreaks. As such, scabies should be repositioned as a public health priority that demands sustained policy attention, resource allocation, and multi‐sectoral collaboration.

## Author Contributions

**Hemayet Hossain:** conceptualization, methodology, software, data curation, investigation, writing – original draft, writing – review and editing. **Md. Al Muktadir:** methodology, software, data curation, investigation, writing – original draft. **Snigdha Sharmin Binte Sayeed:** methodology, data curation, investigation. **Sojib Ahmed:** methodology, software, data curation, investigation. **Md. Hasan Ali:** methodology, software, data curation, investigation. **Khadiza Akter Brishty:** methodology, software, data curation, investigation. **Md. Shahidur Rahman Chowdhury:** methodology, data curation, investigation. **Md. Mahfujur Rahman:** conceptualization, methodology, software, data curation, investigation, supervision, writing – original draft, writing – review and editing. [Correction added on 16 May 2026, after first online publication: Author Contributions section has been modified.]

## Funding

The authors have nothing to report.

## Conflicts of Interest

The authors declare no conflicts of interest.

## Transparency Statement

The lead author Md. Mahfujur Rahman affirms that this manuscript is an honest, accurate, and transparent account of the study being reported; that no important aspects of the study have been omitted; and that any discrepancies from the study as planned (and, if relevant, registered) have been explained.

## Data Availability

The data that support the findings of this study are available from the corresponding author upon reasonable request.
