# Correction to “Scabies Outbreak in Pediatric Populations of Bangladesh: A Perspective on Therapeutic Management, Risk Factors, and Public Health Implications”

**DOI:** 10.1002/hsr2.72538

**Published:** 2026-05-18

H. Hossain, M. A. Muktadir, S. S. Binte Sayeed, et al., “Scabies Outbreak in Pediatric Populations of Bangladesh: A Perspective on Therapeutic Management, Risk Factors, and Public Health Implications,” Health Science Reports 9 (2026): e72450. https://doi.org/10.1002/hsr2.72450.

In the Author Contributions section, the information was incomplete and should be updated as follows:

## Author Contributions

**Hemayet Hossain:** conceptualization, methodology, software, data curation, investigation, writing – original draft, writing – review and editing. **Md. Al Muktadir:** methodology, software, data curation, investigation, writing – original draft. **Snigdha Sharmin Binte Sayeed:** methodology, data curation, investigation. **Sojib Ahmed:** methodology, software, data curation, investigation. **Md. Hasan Ali:** methodology, software, data curation, investigation. **Khadiza Akter Brishty:** methodology, software, data curation, investigation. **Md. Shahidur Rahman Chowdhury:** methodology, data curation, investigation. **Md. Mahfujur Rahman:** conceptualization, methodology, software, data curation, investigation, supervision, writing – original draft, writing – review and editing.

The online version of this article has been corrected accordingly.

We apologize for these errors.

